# *Bellamya purificata* mitigate the impact of *Pomacea canaliculata* through gut microbial changes in rice-snail co-culture system

**DOI:** 10.3389/fmicb.2026.1744121

**Published:** 2026-02-17

**Authors:** Jiaqian Wu, Kangqi Zhou, Liuping Long, Yong Lin, Zhong Chen, Junqi Qin, Xuesong Du, Hui Wei, Hui Luo, Fangdong Yu, Hua Ye, Dangen Gu, Xianhui Pan

**Affiliations:** 1 SEAN Key Laboratory of Comprehensive Exploitation and Utilization of Aquatic Germplasm Resources, Ministry of Agriculture and Rural Affairs, Guangxi Academy of Fishery Sciences, Nanning, China; 2 Key Laboratory of Aquaculture Genetic and Breeding and Healthy Aquaculture, Ministry of Agriculture and Rural Affairs, Guangxi Academy of Fishery Sciences, Nanning, China; 3 Key Laboratory of Freshwater Fish Resources and Development (Ministry of Education), College of Fisheries, Southwest University, Chongqing, China; 4 Pearl River Fisheries Research Institute, Guangzhou, China

**Keywords:** *Bellamya purificata*, ecological invasion, gut microbiota, *Pomacea canaliculata*, rice-snail co-culture system

## Abstract

The rice–snail coculture system represents a resource-efficient and high-yielding agroecological model. However, invasion by the exotic species *Pomacea canaliculata* disrupts its ecological equilibrium. This study examined the influences of *P. canaliculata* on the Dietary Composition and microbiota of the native snail *Bellamya purificata* under monoculture (control) and co-culture (invasion stress) conditions during 30-day and 60-day cultivation periods. Results indicate that both the cultivation condition and duration significantly modified the Dietary Composition of *B. purificata* at the genus level. At 30 days, the co-cultured *B. purificata* group exhibited no significant difference (*p* > 0.05) in gut microbial abundance compared to the mono-culture group, while demonstrating decidedly increased α-diversity and distinctly altered community structure (*p* < 0.05). The relative abundance of *Acinetobacter* was remarkably increased (*p* < 0.05), whereas multiple beneficial bacterial taxa were significantly decreased (*p* < 0.05). Functional prediction analysis revealed dramatically enhanced enrichment of neurodegenerative disease pathways (*p* < 0.05) but considerably reduced enrichment in immune disease pathways and signal molecule interaction pathways (*p* < 0.05). At 60 days, comparative analysis between *B. purificata* co-culture and mono-culture groups revealed no significant differences in gut microbial abundance, α-diversity, and community structure (*p* > 0.05). However, the relative abundances of the phyla *Bacteroidota*, *Actinobacteriota*, and *Planctomycetota* were appreciably upregulated (*p* < 0.05). Genera including *Aeromonas* and *Cloacibacterium* also exhibited notably increased relative abundances (*p* < 0.05). Conversely, the relative abundances of genera such as *Enterobacter* and *Enterococcus* were notably downregulated (*p* < 0.05). Functional prediction analysis further demonstrated greatly enhanced enrichment in cellular motility pathways and cell community-prokaryotic pathways (*p* < 0.05). These results indicate that although stress from *P. canaliculata* exerted a significant short-term negative impact on *B. purificata*, regulatory adaptations through gut microbial changes attenuated this adverse effect following prolonged exposure.

## 1 Introduction

*Bellamya purificata*, belongs to the genus *Bellamya* within the family Viviparidae, which mainly inhabits freshwater environments such as lakes, rivers, canals, and ponds (Li K. et al., 2024). Renowned for its delicious meat, distinctive flavor, and high nutritional value (Luo et al., 2022), it plays a crucial role in China’s economy (Wu et al., 2024). Recent years have witnessed a substantial surge in the commercial sales of Liuzhou Luosifen rice noodles (Total output value exceeds 75 billion RMB). However, the supply of *B. purificata*—the essential ingredient for this industry—primarily relies on wild harvesting, resulting in inconsistent quality and smaller-sized specimens. To ensure a quality-assured and sustainable supply chain for industrial raw materials, developing standardized aquaculture techniques for *B. purificata* has become an urgent priority.

Rice-snail co-culture represents a resource-conserving and high-efficiency aquaculture model, where rice plants provide shade for snails while organic detritus and algae in paddies serve as snail feed. Concurrently, snail excrement functions as natural fertilizer to enhance rice growth, establishing a mutualistic relationship. Currently predominant in China, this integrated system demonstrates robust development momentum in Guangxi, Jiangxi, and Fujian provinces with significant application prospects (Mao et al., 2023). However, the invasion of the exotic species *P. canaliculata* disrupts this ecological equilibrium. It not only gnaws on rice seedlings, causing substantial yield losses (Lv et al., 2024), competes with native snails for food and spatial resources, and preys on their juveniles, but its secretions and excretions also significantly alter the aquatic microenvironment. These alterations, such as elevated nitrogen and phosphorus levels leading to eutrophication, impose physiological stress on native snail populations, which in turn reduces their survival rates (Liu, 2021).

The intestinal tract represents the largest and most complex ecosystem within an organism, playing critical roles in digestion, nutrient absorption, energy metabolism, and immune defense (Li, 2023). As essential components of this system, the gut microbiota exhibit intimate associations with host growth, development, and health status, serving as ideal biomarkers of physiological conditions (Hou et al., 2025). Current research has extensively employed intestinal microbiome analysis to evaluate polyculture systems in aquatic species, including fish, shrimp, and mollusks. Zheng et al. (2025) showed that different co-culture models significantly alter the microbial community structures in both the intestinal tract and environment of *Takifugu rubripes.* Zan et al. (2025) observed distinct genus-level variations in the gut microbiota of *Procambarus clarkii* across rice-crayfish co-culture, pond monoculture, and pond polyculture systems. Similarly, Li et al. (2025a) revealed that polyculturing *Carassius auratus gibelio* in rice-crab integrated systems enhances the microbial diversity in the gut of *Eriocheir sinensis*.

Current studies demonstrate that *P. canaliculata* exhibits superior environmental adaptability—including broad trophic plasticity, drought resistance, acid–base tolerance, and pesticide resistance—compared to native snail species, thereby gaining competitive dominance in ecological niches. Its invasion in shared habitats dramatically reduces native snail survival rates (Fang, 2017; Liu et al., 2021; Li Q. F. et al., 2024; Dai et al., 2025), yet the underlying mechanisms remain elusive. To address this gap, this study employs a comparative analysis of gut microbiota composition and diversity in *B. purificata* before and after *P. canaliculata* invasion within integrated rice-snail co-culture systems. This approach aims to elucidate the mechanistic impacts of the invasive species on native snails, thereby informing targeted invasion control strategies and facilitating the development of ecologically sustainable rice-snail co-culture models.

## 2 Materials and methods

### 2.1 Sample collection and DNA extraction

*B. purificata* (body mass: 1.85 ± 0.56 g; shell height: 20.0 ± 2.11 mm; shell width: 14.82 ± 1.35 mm) and *P. canaliculata* (body mass: 6.55 ± 1.72 g; shell height: 32.68 ± 3.14 mm; shell width: 26.79 ± 3.06 mm) were co-cultured at the rice-snail demonstration base in Ligao Town, Liujiang District, Liuzhou City, Guangxi Province (23.37°N, 111.29°E). The net cages (1 m × 1 m × 1 m) were fully enclosed in structure, with an openable zipper on the top and a base that could be inserted into the sediment for fixation. They were arranged in the paddy field in a layout of 3 columns × 6 rows, totaling 18 cages, with a spacing of 1 meter between both columns and rows.

Six experimental groups were established: *B. purificata* monoculture (BI-30/60), *P. canaliculata* monoculture (PI-30/60), and co-culture with *B. purificata* (BM-30/60) and *P. canaliculata* (PM-30/60) at a 5:1 biomass ratio (BM: PM). Each group was set up with three biological replicates. The monoculture replicates contained 30 individuals of either *B. purificata* or *P. canaliculata*, whereas each replicate in the co-culture group consisted of 25 *B. purificata* and 5 *P. canaliculata* (Liu M. et al., 2025).

Sampling occurred at 30 and 60 days post-co-culture initiation following 24-h starvation to minimize intestinal food residues. To minimize bias caused by individual variation, this experiment employed a pooled sample strategy. Five pooled sequencing samples were prepared for each group. Each pooled sample was created by randomly selecting 10 individuals and combining equal amounts of their DNA samples.

Under aseptic conditions, shells were disinfected with 75% ethanol before extracting soft tissues. Tissues were rinsed thrice with ice-cold sterile 1 × PBS, and intestinal dissection was performed on sterile Petri dishes maintained on ice using sterile instruments. Whole intestinal segments were washed three times with sterile PBS, with luminal contents separately scraped using sterile surgical blades into 4% formaldehyde for dietary analysis. Residual intestinal tissues were homogenized in sterile centrifuge tubes using a Tissuelyser-LT (QIAGEN, Shanghai, China). Microbial DNA was extracted from homogenate using HiPure Stool DNA Kits (Magen, Guangzhou, China) according to manufacturer protocols. All extracts were stored at −80 °C prior to library construction.

### 2.2 Library construction and sequencing

The V3-V4 region of the 16S rRNA gene was amplified by PCR using universal primers 341F (5′-CCTACGGGNGGCWGCAG-3′) and 806R (5′-GGACTACHVGGGTATCTAAT-3′) (Guo et al., 2017). Amplifications were performed in a 50 μL reaction volume containing 10 μL of 5 × Q5® Reaction Buffer, 10 μL of 5 × Q5® High GC Enhancer, 1.5 μL of 2.5 mM dNTPs, 1.5 μL of each primer (10 μM), 0.2 μL of Q5® High-Fidelity DNA Polymerase, and 50 ng of template DNA. All PCR reagents were obtained from New England Biolabs, United States. The thermal cycling conditions consisted of an initial denaturation at 95 °C for 5 min; followed by 30 cycles of denaturation at 95 °C for 1 min, annealing at 60 °C for 1 min, and extension at 72 °C for 1 min; with a final extension at 72 °C for 7 min. Amplified products were pooled, purified, and quantified using a QuantiFluor™ fluorometer (Promega, Beijing, China). Purified amplicons were sequenced on the Illumina HiSeq 2500 platform using a paired-end strategy (PE250) according to the manufacturer’s standard protocols. Raw sequencing reads have been deposited into the NCBI Sequence Read Archive database under accession number PRJNA778015.

### 2.3 Quality control and read assembly

To obtain high-quality clean reads, raw reads containing over 10% unknown nucleotides (N) or with fewer than 50% of bases having a Phred quality score (Q-score) > 20 were removed using FASTP (Chen et al., 2018a). Paired-end clean reads were merged into raw tags using FLASH (v1.2.11) with a minimum overlap of 10 bp and a maximum mismatch rate of 2% (Salzberg, 2011). Noisy sequences within raw tags were filtered under specific conditions using the QIIME (v1.9.1) pipeline to generate high-quality clean tags (Caporaso et al., 2010). Briefly, raw tags were truncated starting from the position of the first low-quality base (default quality score ≤3) where the length of a continuous low-quality segment reached the threshold (default length: 3). Tags where the longest contiguous stretch of high-quality bases was shorter than 75% of the total tag length were discarded. Clean tags were then compared against the reference database^1^ to perform reference-based chimera detection using the UCHIME algorithm (Knight, 2011). All chimeric tags were removed to obtain effective tags for downstream analysis.

### 2.4 Statistical analysis

Algae and zooplankton within Dietary Composition were identified and quantified via microscopic examination. Given that this study utilizes pooled samples to evaluate average inter-group effects and focuses primarily on community-level responses at higher taxonomic ranks, we employed the OTU-based clustering approach. Specifically, high-quality sequencing reads were clustered into operational taxonomic units (OTUs) at 97% sequence similarity using the UPARSE pipeline (v9.2.64) for downstream intestinal microbiota analyses. The most abundant sequence within each OTU was designated as its representative sequence. Venn diagrams (VennDiagram v1.6.16) and UpSet plots (UpSetR v1.3.3) were employed to visualize unique and shared OTUs across experimental groups. Representative sequences were classified against the SILVA database using the RDP Classifier (v2.2) with a naive Bayesian model at a confidence threshold of 80%. Taxonomic abundance was visualized with Krona (v2.6) (Ondov et al., 2011). Community composition was illustrated through stacked bar plots (ggplot2 v2.2.1) and ternary diagrams (ggtern v3.1.0). Alpha diversity indices (including Sobs, Chao1, ACE, Shannon and Simpson) were calculated in QIIME, with rarefaction curves and rank-abundance plots generated using ggplot2. Inter-group alpha diversity comparisons utilized Welch’s *t*-tests (vegan v2.5–3), while multi-group differences were assessed by Kruskal-Wallis tests with Tukey’s HSD post-hoc analysis. Beta diversity was evaluated through Principal Coordinates Analysis (PCoA) of Bray-Curtis distances (vegan v2.5-3), with statistical significance determined by PERMANOVA (adonis function). Differential abundance testing for indicator species identification was performed using Welch’s *t*-tests (vegan), while biomarker screening implemented the labdsv package (v2.0-1). Functional potential was predicted via KEGG pathway analysis of OTUs using PICRUSt2 (v2.1.4).

## 3 Results

### 3.1 Statistical analysis of growth and survival

At 30 days of cultivation, the average body weights of *B. purificata* in the monoculture and co-culture groups were 2.85 g and 2.05 g, respectively, with average shell heights of 26.32 mm and 19.02 mm and survival rates of 96.7 and 78.9%. For *P. canaliculata*, the average body weights in the monoculture and co-culture groups were 10.48 g and 9.17 g, respectively, with average shell heights of 29.57 mm and 28.47 mm and survival rates of 97.8 and 93.3%.

At 60 days of cultivation, the average body weights of *B. purificata* in the monoculture and co-culture groups were 3.84 g and 2.45 g, respectively, with average shell heights of 27.82 mm and 23.11 mm and survival rates of 91.1 and 68%. For *P. canaliculata*, the corresponding values were14.41 g and 11.79 g for average body weight, 32.35 mm and 30.15 mm for average shell height, and 92.2 and 81.1% for survival rate.

These results indicate that, at both 30 and 60 days of cultivation, the body weight, shell height, and survival rate of both *B. purificata* and *P. canaliculata* in the co-culture group were significantly lower than those in their respective monoculture groups (Table 1).

**Table 1:** Growth performance and survival rate of *Pomacea canaliculata* and *Bellamya purificata.*

Group	Body weights (g)	Shell heights (mm)	Survival rates (%)
*Bellamya purificata*	1.85 ± 0.56	14.82 ± 1.35	100
BI-30	2.85 ± 0.86^a^	26.32 ± 1.49^a^	96.67 ± 1.00^a^
BM-30	2.05 ± 0.50^c^	19.02 ± 1.37^b^	78.89 ± 1.53^c^
BI-60	3.84 ± 1.16^a^	27.82 ± 1.62^a^	91.11 ± 0.58^a^
BM-60	2.45 ± 0.44^c^	23.11 ± 1.39^b^	68.00 ± 2.00^c^
*Pomacea canaliculata*	6.55 ± 1.72	26.79 ± 3.06	100
PI-30	10.48 ± 2.76^a^	29.57 ± 3.38^c^	97.78 ± 1.15^a^
PM-30	9.17 ± 2.41^b^	28.47 ± 3.25^c^	93.33 ± 1.00^ab^
PI-60	14.41 ± 3.79^a^	32.35 ± 3.69^d^	92.22 ± 1.53^a^
PM-60	11.79 ± 3.10^b^	30.15 ± 3.44^c^	81.11 ± 2.52^b^

### 3.2 Analysis of gut food composition

#### 3.2.1 Analysis of gut food composition

**Figure 1:**
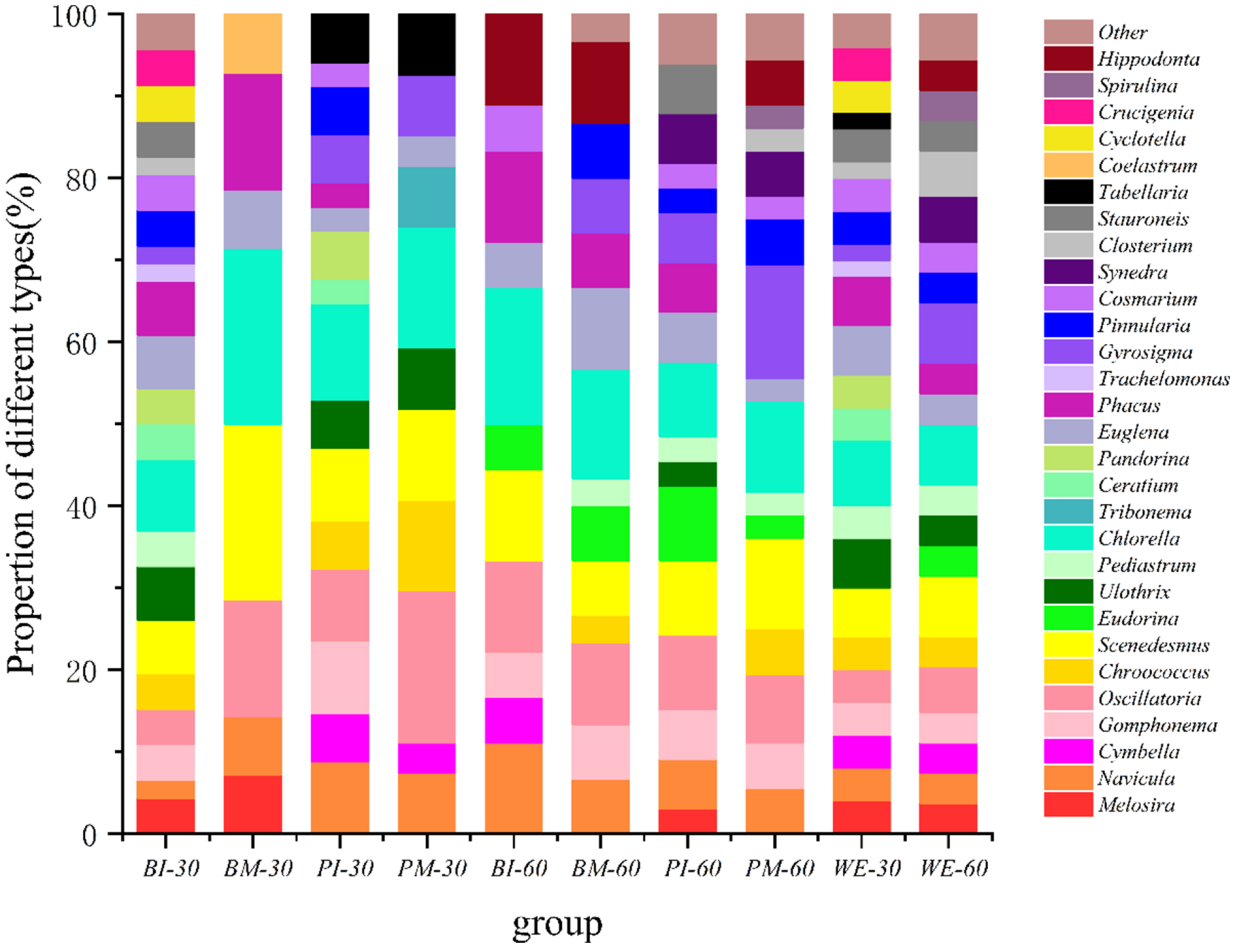
Genus-level composition of dietary composition in *Pomacea canaliculata* and *Bellamya purificata* with environmental sample profiles. Sample codes denote species, culture condition, and time (days): BI, monoculture of *B. purificata*; PI, monoculture of *P. canaliculata*; BM, co-cultivated *B. purificata*; PM, co-cultivated *P. canaliculata*; WE, water environment. Numeric suffix indicates cultivation time (30 or 60 days).

Microscopic observation and statistical analysis of genus-level food composition in intestinal samples from *B. purificata* and *P. canaliculata*, as well as the surrounding aquatic environment, are presented in Figure 1. The results reveal diversified proportional distributions of gut food composition across experimental groups. The BI-30 group’s dietary composition was primarily composed of *Chlorella, Scenedesmus, Ulothrix, Euglena*, and *Phacus*, constituting approximately 34.78% of the total. In the BM-30 group, *Chlorella, Scenedesmus, Phacus*, and *Oscillatoria* dominated the intestinal food at 71.43%. The BI-60 group exhibited a dietary profile mainly comprising *Chlorella, Oscillatoria, Scenedesmus, Navicula, Phacus*, and *Hippodonta*, accounting for 72.22%. *Chlorella, Oscillatoria, Hippodonta,* and *Euglena* formed the principal components (43.33%) in the BM-60 group. The PI-30 group’s gut food Composition was dominated by *Chlorella, Scenedesmus, Oscillatoria, Navicula,* and *Gomphonema* at 47.06%, while the PM-30 group primarily contained *Scenedesmus, Chlorella, Gyrosigma*, and *Gomphonema* (55.56%). For the PI-60 group, *Chlorella, Oscillatoria, Scenedesmus,* and *Eudorina* constituted 36.36% of the diet, whereas the PM-60 group featured *Gyrosigma, Chlorella, Scenedesmus*, and *Oscillatoria* as major components (44.44%). Environmental samples showed that the WE-30d composition was principally *Chlorella, Scenedesmus, Euglena, Phacus,* and *Ulothrix* (32.00%), while the WE-60d sample was dominated by *Chlorella, Scenedesmus, Gyrosigma, Oscillatoria, Closterium,* and *Synedra* at 38.89%.

#### 3.2.2 α-diversity analysis of gut food composition

This study used α-diversity indices to analyze the species richness and diversity of food composition in *B. purificata*, *P. canaliculata*, and their aquaculture water environment. As shown in the results, at 30 days of cultivation, the Sob, Simpson, and Shannon indices of gut food composition in the monoculture groups of both snail species were significantly higher than those in their corresponding co-culture groups. By 60 days, no significant differences were observed in these indices between monoculture and co-culture groups.

**Figure 2:**
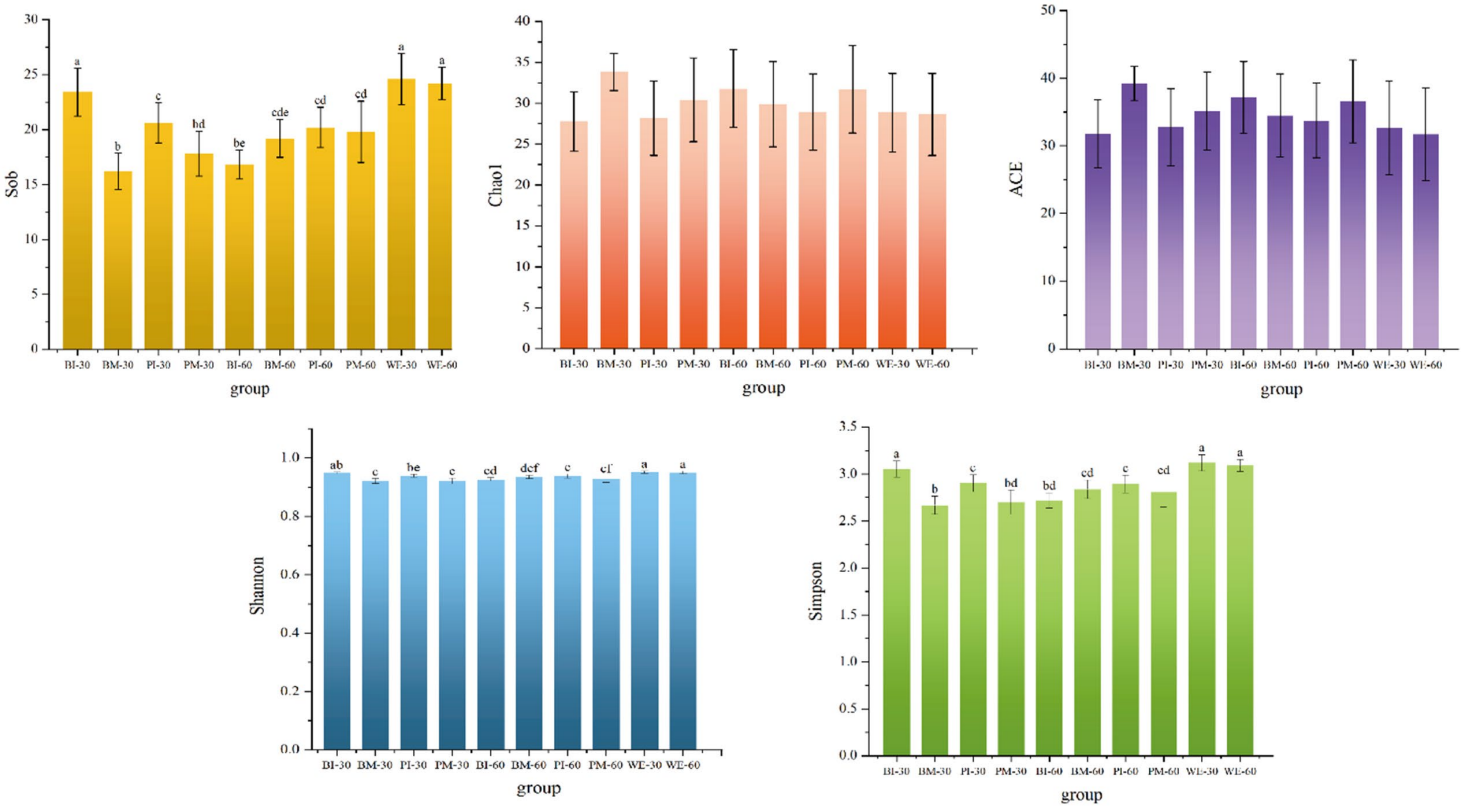
α-diversity of analysis of gut food composition.

Comparing different cultivation periods, in *B. purificata*, the Sob, Simpson, and Shannon indices of gut food were significantly higher at 30 days than at 60 days in the monoculture group, whereas the opposite trend was observed in the co-culture group. For *P. canaliculata*, no significant differences were detected between 30 and 60 days in either monoculture or co-culture groups. Furthermore, no significant differences were found in any α-diversity indices of the water environment between 30 and 60 days, and neither the Chao1 index nor the ACE index showed significant differences among all groups (Figure 2).

#### 3.2.3 β-diversity analysis of gut food composition

**Figure 3:**
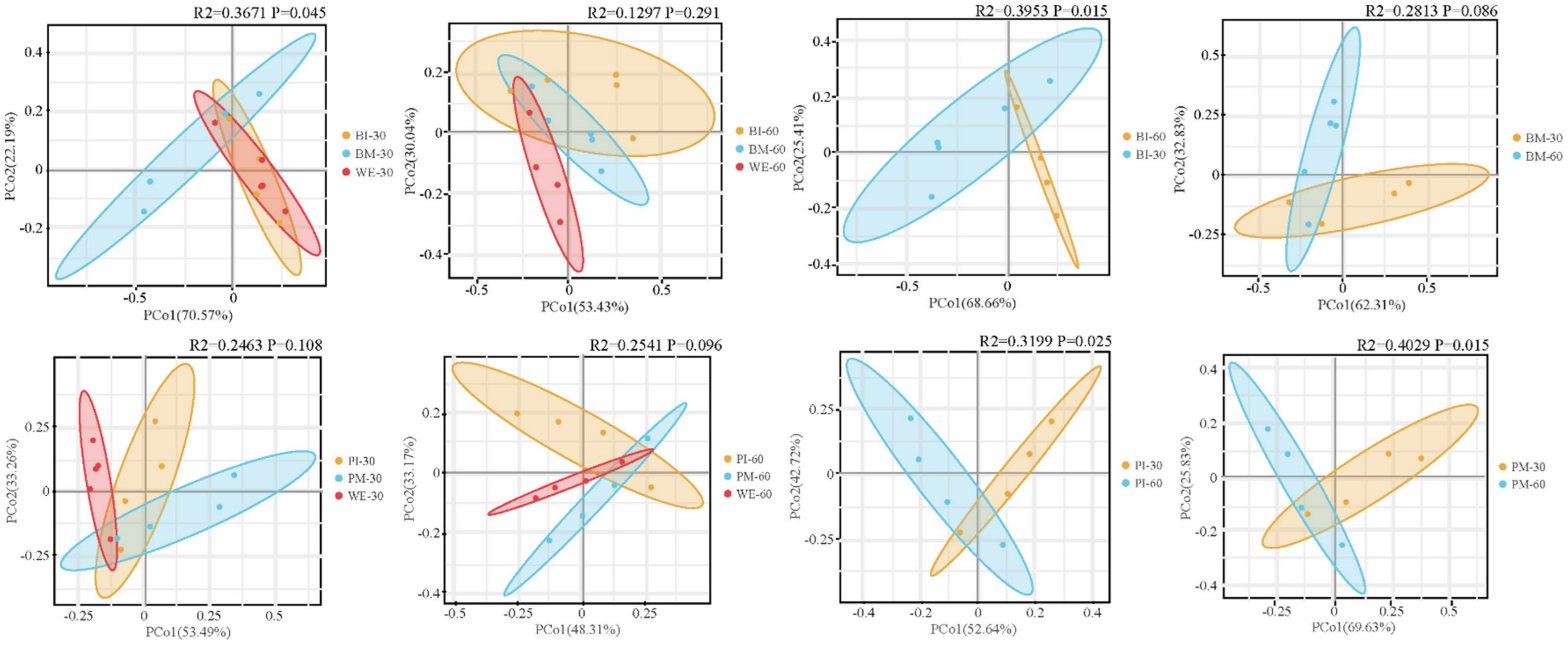
β-diversity of analysis of gut food composition.

Beta diversity analysis was conducted on the food composition of *B. purificata*, *P. canaliculata*, and their aquatic environment. The results showed that for *B. purificata*, the gut food community structure differed significantly between the monoculture and co-culture groups at 30 days of cultivation, while no significant difference was observed at 60 days. In *P. canaliculata*, no significant differences were found between monoculture and co-culture groups at either 30 or 60 days. Furthermore, when comparing the same groups across different cultivation periods, both the monoculture and co-culture groups of the two snail species exhibited significant differences in gut food community structure between 30 and 60 days (Figure 3).

### 3.3 Analysis of gut microbiota composition

#### 3.3.1 Analysis of 16S rRNA high-throughput sequencing results

**Figure 4:**
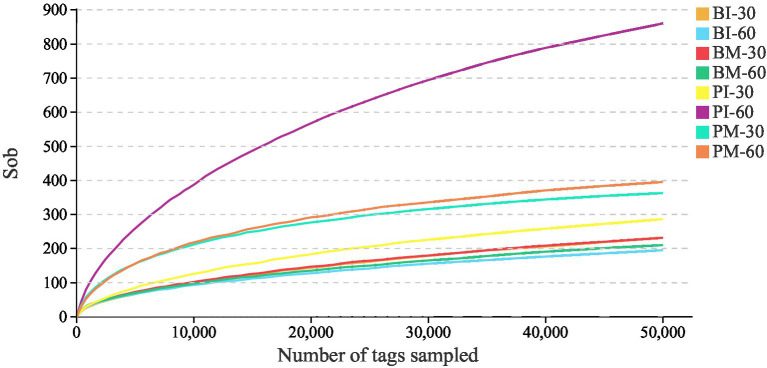
Species saturation rarefaction curves of gut microbiota in *Pomacea canaliculata* and *Bellamya purificata*.

A total of 4,458,267 raw reads were obtained from sequencing all samples. Quality control analysis using USEARCH yielded 3,849,920 high-quality effective tags, with all samples retaining over 81% of their original reads, demonstrating excellent sequencing efficiency. The UPARSE (v9.2.64) pipeline was employed to cluster the effective tags into 16,178 operational taxonomic units (OTUs) at a 97% similarity threshold (Table 2). The rarefaction curves plateaued, suggesting that the sequencing depth was sufficient to capture the majority of microbial diversity present in the samples (Figure 4).

**Table 2:** High-throughput sequencing results of gut microbiota in *Pomacea canaliculata* and *Bellamya purificata*.

Group	Effective tags	Clean tags	OTUs	goods_coverage
BI-30	93,083	107,151	289	99.851
BI-60	91,572	108,648	232	99.882
BM-30	97,227	114,544	284	99.852
BM-60	95,299	111,985	259	99.873
PI-30	102,536	115,904	355	99.872
PI-60	95,595	104,515	951	99.887
PM-30	95,823	105,398	411	99.898
PM-60	98,849	108,146	455	99.889

#### 3.3.2 α-diversity analysis of gut microbiota in *Pomacea canaliculata* and *Bellamya purificata*

To evaluate the impact of *P. canaliculata* introduction on the gut microbial richness and diversity of *B. purificata*, α-diversity analysis was conducted. The results showed that the Good’s coverage index of each group exceeded 99%, indicating that the sequencing results sufficiently represented the actual composition of the samples. After 30 days of rearing, the co-culture group of *B. purificata* showed no significant differences in microbial richness indices (Sobs, Chao1, ACE) compared to the monoculture group, but exhibited dramatically increased diversity indices (Shannon and Simpson; *p* < 0.05). Similarly, in *P. canaliculata*, the co-culture group showed no significant differences in Sobs, Chao1, ACE, or Simpson indices compared to the monoculture group, but a marked increase in the Shannon index was observed (p < 0.05). The above findings demonstrated that *P. canaliculata* invasion altered the gut diversity and abundance levels of *B. purificata*.

**Figure 5:**
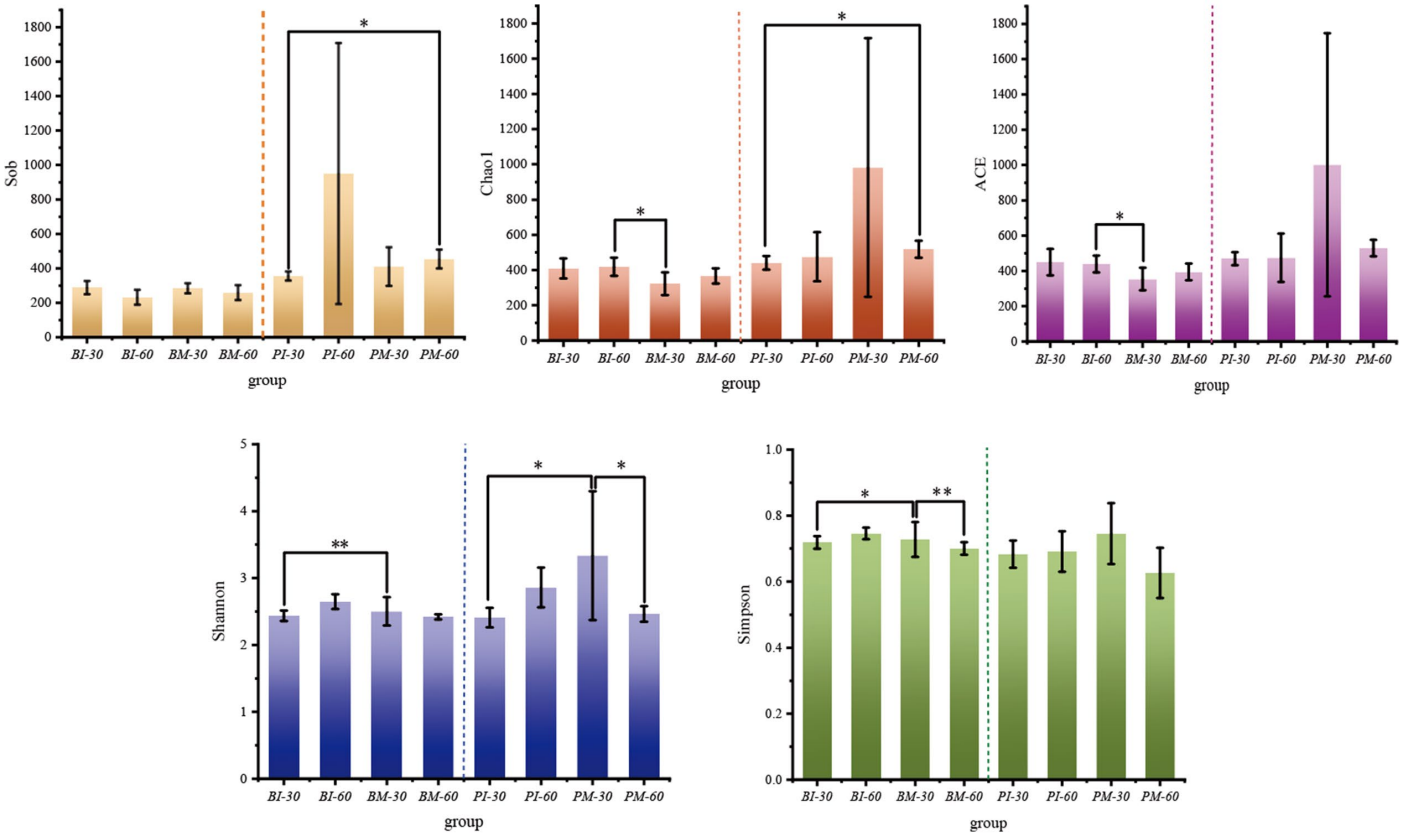
α-diversity of gut microbiota in *Pomacea canaliculata* and *Bellamya purificata.*

After 60 days of rearing, neither the co-cultured *B. purificata* nor *P. canaliculata* showed significant differences in microbial richness or diversity indices compared to their respective monoculture groups. When comparing the monoculture groups of *B. purificata* and *P. canaliculata* between 30 and 60 days, no meaningful differences were observed in any gut microbial richness or diversity indices. In the co-culture groups, microbial richness indices did not differ significantly between 30 and 60 days; however, the Simpson index of *B. purificata* and the Shannon index of *P. canaliculata* were considerably higher at 30 days than at 60 days. This suggests that long-term *P. canaliculata* invasion exerts deeper impacts on native species (Figure 5).

#### 3.3.3 β-diversity analysis of gut microbiota in *Pomacea canaliculata* and *Bellamya purificata*

**Figure 6:**
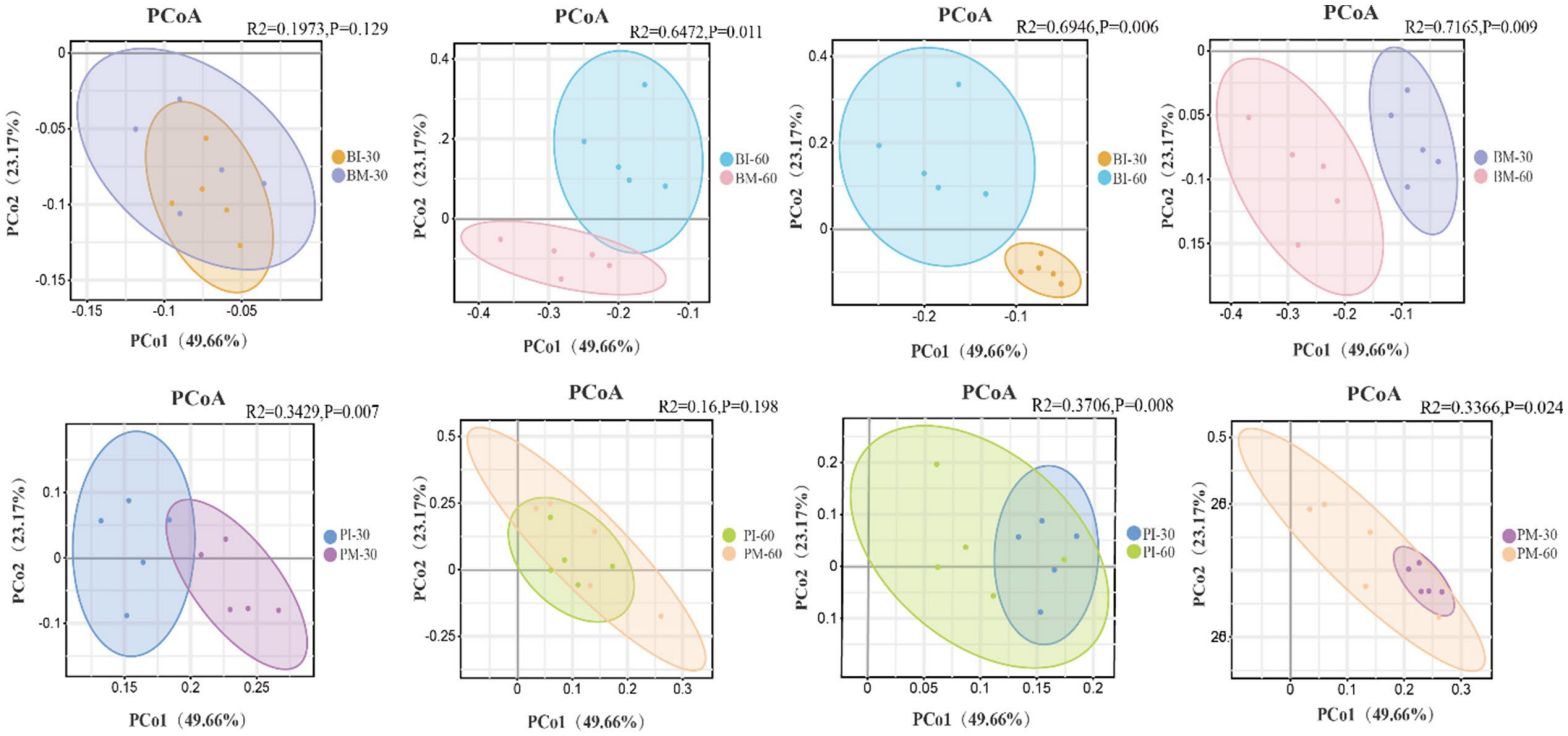
β-diversity of gut microbiota in *Pomacea canaliculata* and *Bellamya purificata.*

As shown in the figure, Principal Coordinates Analysis (PCoA) based on Bray-Curtis distances and Adonis tests revealed no significant difference (*p* > 0.05) in gut microbial community structure between monoculture and co-culture groups of *B. purificata* at 30 days of cultivation. However, remarkable variation was observed at 60 days (*R*^2^ = 0.6472, *p* = 0.011). For *P. canaliculata*, major difference was found between monoculture and co-culture groups at 30 days (*R*^2^ = 0.3429, *p* = 0.008), but not at 60 days (*p* > 0.05). The microbial community structures of both the monoculture and co-culture groups of each species differed significantly (*p* < 0.05) between the 30-day and 60-day cultivation periods (Figure 6).

#### 3.3.4 Analysis of gut microbial composition in *Pomacea canaliculata* and *Bellamya purificata*

**Figure 7:**
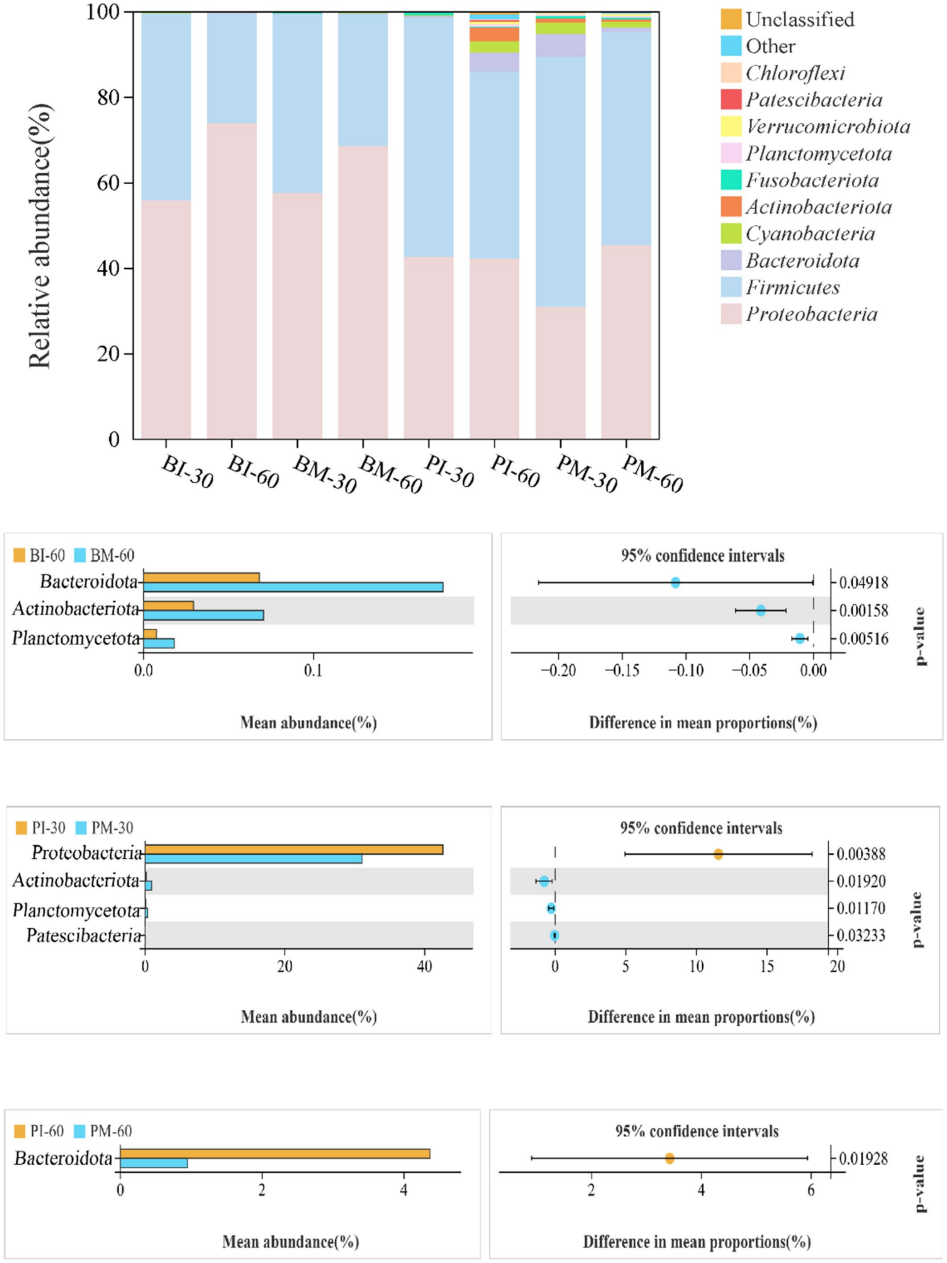
Phylum-level composition and significant differences in gut microbiota of *Pomacea canaliculata* and *Bellamya purificata.*

At the phylum level, analysis of the gut microbiota in *P. canaliculata* and *B. purificata* identified 10 phyla across all groups, including *Proteobacteria*, *Firmicutes*, *Bacteroidota*, *Cyanobacteria*, and *Actinobacteriota*, among which *Proteobacteria* and *Firmicutes* were the absolutely dominant phyla. Welch’s *t*-test showed no significant differences in the relative abundance of gut microbiota at the phylum level between monoculture and co-culture groups of *B. purificata* at 30 days (*p* > 0.05). In *P. canaliculata* at 30 days, the relative abundance of the *Proteobacteria* phylum was substantially reduced in the co-culture group compared to the single-culture group (*p* < 0.05), while the abundances of the *Actinobacteria*, *Ascomycota*, and *Bacteroidota* phyla were significantly elevated (*p* < 0.05). At 60 days, the co-culture group of *B. purificata* exhibited considerably higher relative abundances of *Bacteroidota*, *Actinobacteriota*, and *Planctomycetota* than the monoculture group (*p* < 0.05), while the co-culture group of *P. canaliculata* had a dramatically lower abundance of *Bacteroidota* (*p* < 0.05) (Figure 7).

**Figure 8:**
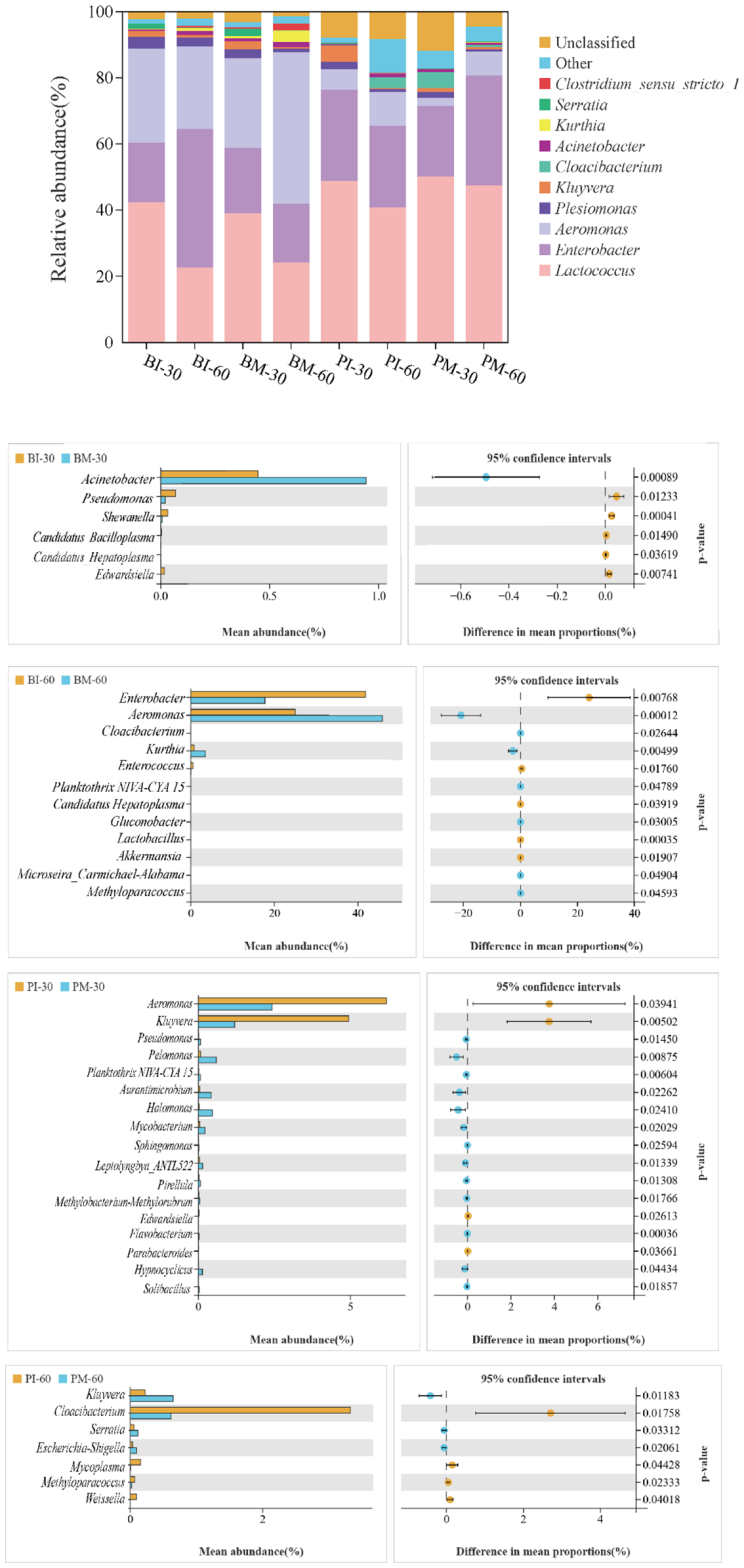
Genus-level composition of gut microbiota in *Pomacea canaliculata* and *Bellamya purificata.*

Analysis of gut microbiota at the genus level in *B. purificata* and *P. canaliculata* identified 12 genera across all groups, including *Lactococcus*, *Enterobacter*, *Aeromonas*, *Plesiomonas*, *Kluyvera*, and *Cloacibacterium*. Among these, *Lactococcus*, *Enterobacter*, and *Aeromonas* consistently ranked as the top three genera in relative abundance across all groups, representing the dominant microbiota in both species (Figure 8).

At day 30, the co-culture group of *B. purificata* exhibited a notably higher relative abundance of *Acinetobacter* but significantly lower abundances of *Acinetobacter*, *Shewanella*, *Edwardsiella*, *Candidatus_Bacilloplasma*, and *Candidatus_Hepatoplasma* compared to the monoculture group. In *P. canaliculata*, the co-culture group showed markedly higher relative abundances of 13 genera including *Acinetobacter*, *Pelomonas*, and *Planktothrix*_NIVA-CYA_15, along with dramatically lower abundances of *Aeromonas*, *Kluyvera*, *Edwardsiella*, and *Parabacteroides* relative to the monoculture group.

By day 60, the co-culture group of *B. purificata* displayed greatly increased relative abundances of 12 genera (including *Aeromonas*, *Cloacibacterium*, and *Kurthia*) but notably decreased abundances of *Enterobacter*, *Enterococcus*, *Candidatus Hepatoplasma*, *LactoBacillus*, and *Akkermansia* compared to the monoculture group. For *P. canaliculata*, the co-culture group showed considerably higher relative abundances of *Kluyvera*, *Serratia*, and *Escherichia-Shigella* yet significantly lower abundances of *Cloacibacterium*, *Mycoplasma*, *Methyloparacoccus*, and *Weissella* compared to its monoculture group.

#### 3.3.5 Correlation analysis between gut food composition and gut microbiota

**Figure 9:**
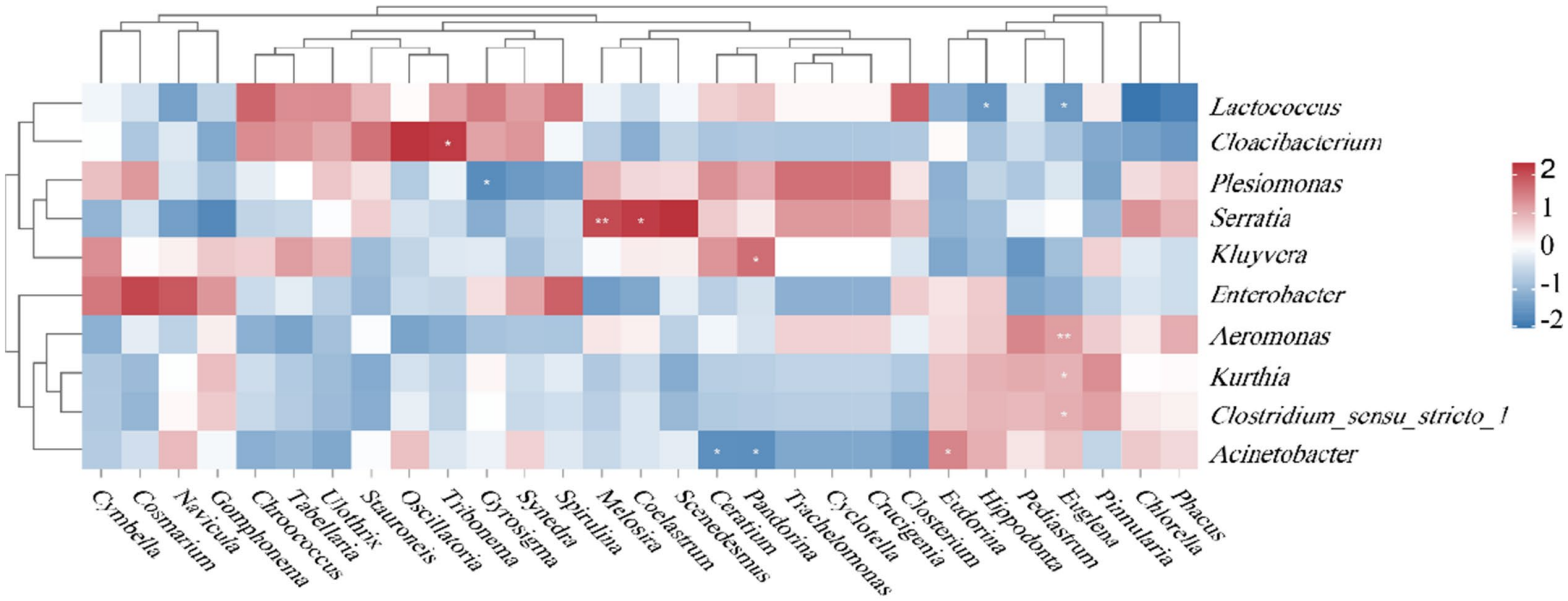
Correlation analysis between gut microbiota and dietary composition in *Bellamya purificata.*

A correlation analysis was conducted at the genus level to assess the relationships between the relative abundance of gut microbiota and that of algae in the dietary composition. The results revealed several significant correlations: *Lactococcus* showed significant negative correlations with *Euglena* and *Hippodonta* (*p* < 0.05), with correlation coefficients of −0.726 and −0.814, respectively; *Cloacibacterium* was dramatically positively correlated with *Tribonema* (*p* < 0.05, *r* = 0.798); *Plesiomonas* exhibited a significant negative correlation with *Gyrosigma* (*p* < 0.05, *r* = −0.779); *Serratia* was significantly positively correlated with *Melosira* and *Coelastrum* (*r* = 0.898 and 0.801, respectively); and *Kluyvera* showed a significant positive correlation with *Pandorina* (*p* < 0.05, *r* = 0.812) (Figure 9).

Additionally, *Aeromonas* was greatly positively correlated with *Euglena* (*p* < 0.05, *r* = 0.918); *Kurthia* was markedly positively correlated with *Euglena* (*p* < 0.05, *r* = 0.789); *Clostridium_sensu_stricto_1* showed a significant positive correlation with *Euglena* (*p* < 0.05, *r* = 0.812); and *Acinetobacter* was appreciably positively correlated with *Eudorina* (*p* < 0.05, *r* = 0.774) but decidedly negatively correlated with *Ceratium* and *Pandorina* (*p* < 0.05, *r* = −0.723 and −0.775, respectively).

#### 3.3.6 Functional analysis of intestinal microbiota in *Pomacea canaliculata* and *Bellamya purificata*

PICRUSt2 analysis revealed that the predicted functional genes of gut microbiota from all samples were annotated to 6 Level1 pathways and 28 Level2 pathways (mean relative abundance >1%) in the KEGG database. Results of Level1 pathways indicated that functional genes were predominantly enriched in Metabolism, Genetic Information Processing, Cellular Processes, Environmental Information Processing, Human Diseases, and Organismal Systems. Metabolism-related pathways accounted for approximately 80% of enriched functional genes across all groups, suggesting that the primary function of gut microbiota in both *P. canaliculata* and *B. purificata* is to facilitate the digestion and metabolism of various substances within the gut. Analysis of Level2 pathways showed that genes were mainly enriched in carbohydrate metabolism, metabolism of cofactors and vitamins, amino acid metabolism, metabolism of terpenoids and polyketides, metabolism of other amino acids, xenobiotic biodegradation and metabolism, xenobiotics biodegradation and metabolism, lipid metabolism, energy metabolism, replication and repair, and glycan biosynthesis and metabolism under the Metabolism category.

**Figure 10:**
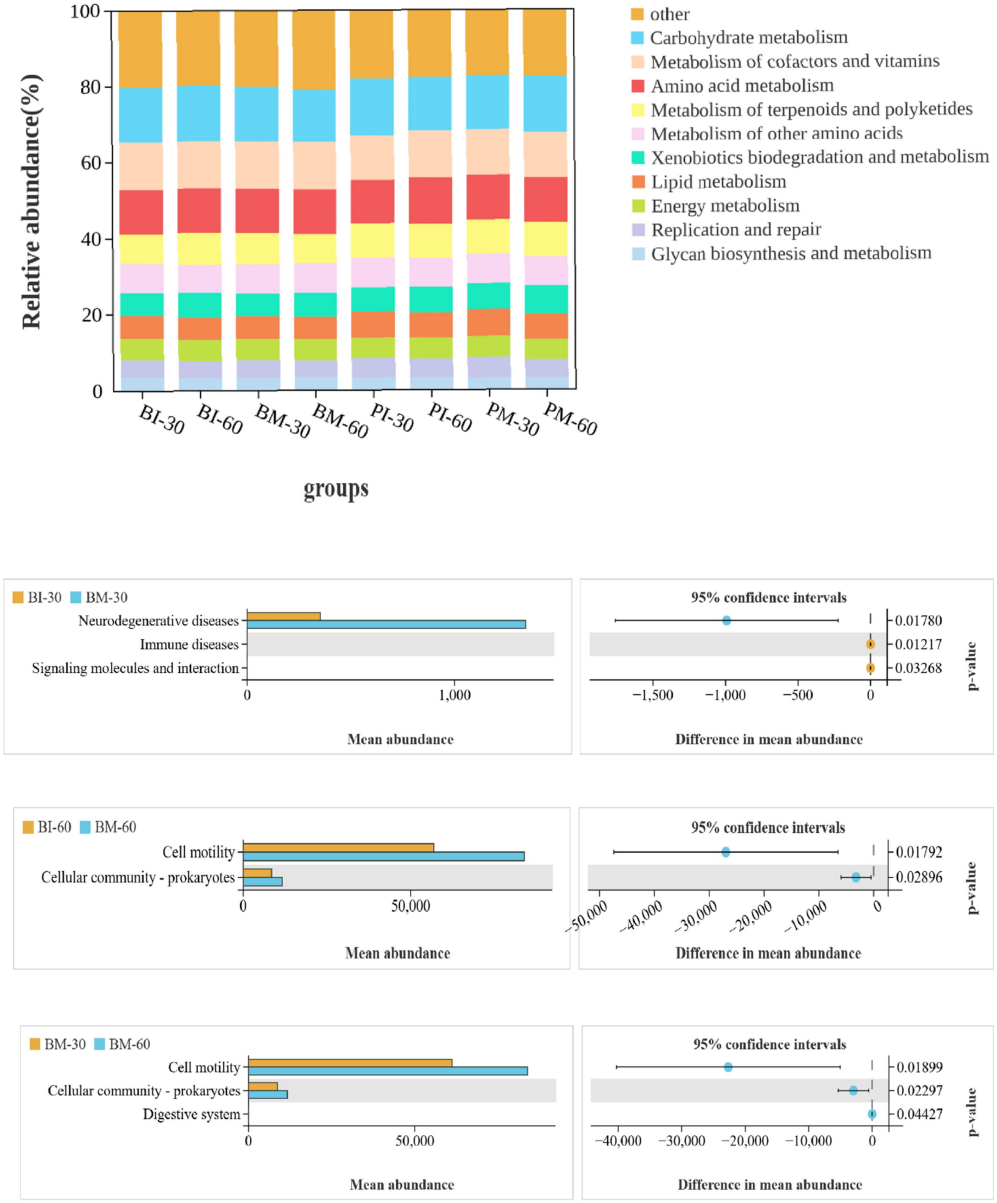
Level 2 functional predictions of gut microbial communities in *Pomacea canaliculata* and *Bellamya purificata.*

Differential analysis of functional gene enrichment in Level2 pathways among groups is shown in the corresponding figure. At 30 days of cultivation, the co-culture group of *B. purificata* showed dramatically higher enrichment in pathways related to neurodegenerative diseases (*p* < 0.05), and notably lower enrichment in immune diseases and signaling molecules and interaction pathways compared to the monoculture group. At 60 days, the co-culture group of *B. purificata* exhibited significantly higher enrichment in cell motility and cellular community—prokaryotes pathways than the monoculture group. Furthermore, the co-culture group of *B. purificata* at 60 days showed greatly higher enrichment in cell motility, cellular community—prokaryotes, and digestive system pathways compared to that at 30 days. In contrast, no significant differences (*p* > 0.05) were observed in the enrichment of Level2 pathways between the 30-day and 60-day monoculture groups of *B. purificata* (Figure 10).

## 4 Discussion

### 4.1 Gut food composition of *Bellamya purificata* and *Pomacea canaliculata*

The feeding habits of aquatic animals are influenced by foraging modes, growth stages, physiological activities, and environmental factors. Understanding these trophic dynamics is essential for elucidating ecological relationships between organisms and their environment, particularly regarding their nutritional niches within ecosystems (Han et al., 2013).

In all experimental groups, *Chlorella*, *Scenedesmus*, and *Oscillatoria* constituted predominant components of dietary composition, indicating consistent consumption of these algae by both snail species. This suggests potential interspecific competition for algal resources (Yang et al., 2024). Compared to monoculture groups, *B. purificata* co-culture groups exhibited an increased proportion of *Eudorina* but decreased *Euglena* in their dietary composition. For *P. canaliculata* co-culture groups, elevated proportions of Pinnularia, *Scenedesmus*, *Gyrosigma*, and *Chlorella* were observed alongside reduced proportions of *Phacus*, *Ulothrix*, and *Oscillatoria*. These shifts likely reflect food competition or selective feeding under co-culture conditions (Svanbäck and Bolnick, 2007). Temporal variations revealed distinct foraging preferences: after 60 days compared to 30 days, *B. purificata* showed increased consumption of Kirchneriella and *Eudorina* but decreased *Melosira*, while *P. canaliculata* exhibited reduced *Eudorina* intake. These ontogenetic dietary shifts align with established patterns of feeding plasticity during growth (Bryan et al., 2021; Hüne et al., 2023).

Water environment samples (WE-30/WE-60) demonstrated stable algal composition, with *Chlorella*, *Scenedesmus*, *Euglena*, and *Phacus* dominating both periods. Notably, these algae that were abundant in the water environmental samples consistently comprised a high proportion across all samples. Although *Cyclotella* accounted for 4% of the composition in the WE-30 water sample, it was only detected in the BI-30 culture group. This pattern highlights both the similarities and distinctions between the environmental availability of resources and the dietary composition. Therefore, it can be concluded that both the culture method (monoculture or co-culture) and the duration of culture (30 d or 60 d) had significant effects on the gut dietary composition of *B. purificata* and *P. canaliculata* at the genus level. The shifts in the relative abundance of various genera reflect their feeding preferences and ecological adaptability under different environmental conditions. Furthermore, a close trophic association was observed between the aquatic animals and their surrounding water environment.

### 4.2 Gut food diversity and community structure composition of *Bellamya purificata* and *Pomacea canaliculata*

Based on the α- and β-diversity analyses of the intestinal dietary composition in *B. purificata* and *P. canaliculata*, this study revealed patterns of food resource utilization by these two snail species under different cultivation modes.

At 30 days of cultivation, the monoculture groups of both snail species exhibited significantly higher α-diversity indices compared to their co-culture groups. A significant difference in intestinal food community structure was observed between the monoculture and co-culture groups of *B. purificata*, whereas no such significant difference was found for *P. canaliculata*. Therefore, it is hypothesized that interspecific competition may have led to a compression of their dietary niches (Schulze et al., 2012). *P. canaliculata*, potentially due to its larger body size, may hold a competitive advantage in resource acquisition, or its broader dietary range may contribute to the stability of its gut food community structure (Wang Y. et al., 2025). In contrast, *B. purificata* may have undergone adjustments in its feeding behavior in response to the pressure from interspecific competition (Charette et al., 2024).

At 30 days of cultivation, the monoculture groups of both snail species exhibited notably higher α-diversity indices compared to their co-culture groups. A significant difference in intestinal food community structure was observed between the monoculture and co-culture groups of *B. purificata*, whereas no such marked difference was found for *P. canaliculata*. Therefore, it is hypothesized that interspecific competition may have led to a compression of their dietary niches. *P. canaliculata*, potentially due to its larger body size, may hold a competitive advantage in resource acquisition, or its broader dietary range may contribute to the stability of its gut food community structure. In contrast, *B. purificata* may have undergone adjustments in its feeding behavior in response to the pressure from interspecific competition.

At 60 days of cultivation, no substantial differences were detected in either α-diversity indices or intestinal food community structure between the co-culture and monoculture groups for either snail species. This may be attributed to the fact that, in response to the prolonged stress from *P. canaliculata*, *B. purificata* may have enhanced its utilization of dominant food resources. This behavioral adjustment could have facilitated the avoidance of direct competition and a reduction in niche overlap, ultimately leading to a gut food composition in the co-culture group that approximated its state under monoculture conditions (Duan, 2013).

Under monoculture conditions, *B. purificata* exhibited a trend of decreasing dietary diversity over time, whereas the opposite trend was observed under co-culture conditions. This likely reflects adjustments in its feeding behavior in response to interspecific competitive pressure. In contrast, *P. canaliculata* showed no significant temporal changes in diversity under either monoculture or co-culture conditions, demonstrating greater stability in biotic interaction environments.

The lack of significant differences in the α-diversity of the aquaculture water and in Chao1 and ACE indices among all groups indicates that the observed changes in food composition are more likely attributable to active feeding selection and behavioral adaptation by the snails, rather than to alterations in the available food resource pool itself.

Collectively, these results suggest that *B. purificata* is initially more sensitive to competitive pressure but gradually exhibits dietary adaptation over time, whereas *P. canaliculata* maintains a relatively stable pattern.

### 4.3 Core gut microbiota of *Bellamya purificata* and *Pomacea canaliculata*

Dominant microbial phyla in aquatic organisms predominantly include *Proteobacteria*, *Bacteroidota*, and *Firmicutes* (Shi et al., 2023). *Proteobacteria* encompasses diverse bacteria with functional roles in nutrient metabolism (Feng et al., 2008), immunomodulation (Sugita et al., 1996), and environmental adaptation (Xiang et al., 2022), though many species within this phylum are pathogenic. While *Enterobacteriaceae* can convert non-essential amino acids into essential amino acids or proteins (Ben-Yosef et al., 2010), pathogenic members like *Escherichia* and *Salmonella* may cause diarrhea and enteritis (Janda and Abbott, 2021). Similarly, *Aeromonadaceae* species produce extracellular enzymes enhancing host digestion (Zeng and Yuang, 1999; Koca et al., 2015), yet *Aeromonas veronii* induces intestinal inflammation in fish (Yin, 2022), and *A. hydrophila* causes bacterial septicemia (Sun et al., 2023). *Firmicutes* contribute crucially to host nutritional metabolism and immunoregulation, facilitating dietary energy harvest through carbohydrate fermentation while producing anti-inflammatory compounds that maintain intestinal homeostasis (Säemann et al., 2000; Magne et al., 2020). For instance, *Lactococcus* can modulate lysozyme activity, inhibit pathogen growth, promote mucus secretion, and strengthen the physical barrier function (Paritova et al., 2024), and *Bacillus* species induce antimicrobial peptide production, enhancing mucosal defense (Safarchi et al., 2025).

*Proteobacteria* and *Firmicutes* constituted the predominant phyla in the gut microbiota of *B. purificata* and *P. canaliculata*, representing over 99 and 85% of the total abundance, respectively. At the genus level, *Lactococcus*, *Enterobacter*, and *Aeromonas* collectively accounted for over 85 and 73% in all groups of the two species, respectively. These taxa represent the core microbiota at the phylum and genus levels in both snails, indicating fundamental roles in host metabolic and immune processes.

### 4.4 Impact of *Pomacea canaliculata* stress on gut microbial diversity and community structure in *Bellamya purificata*

The gut tract is one of the most closely connected interfaces between the host and the external environment. Previous studies have shown that the invasion of *P. canaliculata* can occupy the living areas of native species, reduce food abundance, and secrete harmful substances that alter the aquatic environment (such as pH and transparency), leading to disease or death among local snails (Liu M. et al., 2025).

In this study, at 30 days of cultivation, the gut microbial abundance showed no significant difference between co-culture and monoculture groups of *B. purificata*, while the microbial diversity was appreciably higher in co-culture groups. By 60 days, neither microbial abundance nor diversity differed decidedly between co-culture and monoculture groups of *B. purificata*. These results indicate that co-culture did not significantly affect microbial abundance in either snail species, but it transiently increased gut microbial diversity in the short term. However, this effect diminished over time. Studies on yellow catfish (Zhu et al., 2021), *opsariichthys* (Cui et al., 2025), *grass carp* (Xiao et al., 2023) and *kelp grouper* (Li et al., 2015) have generally reported that polyculture can enhance the diversity of intestinal microbiota in aquatic animals. In contrast, our study observed higher microbial diversity in monoculture groups at 60 days, which may be associated with changes in environmental biochemical factors, distinct growth stages, feeding habits, and immune-metabolic adaptations (Li et al., 2015; An, 2024).

Both monoculture and co-culture groups showed a decreasing trend in gut microbial abundance and diversity over time. However, aside from a significant reduction in microbial diversity observed in the co-culture groups at 60 days compared to 30 days, no other differences were statistically significant. This pattern may be attributed to the prolonged consumption of resources in the water environment, which led to a decline in microbial availability in the aquaculture system, thereby reducing gut microbial diversity (Wang et al., 2025).

Notably, no significant difference was observed in the microbial community structure between the monoculture and co-culture groups of *B. purificata* at 30 days, whereas a statistically significant difference emerged by 60 days. This indicates that the stress exerted by *P. canaliculata* on *B. purificata* exhibits a time-dependent effect. The underlying reason may be that prolonged invasion by *P. canaliculata* may alter physicochemical factors of water environment and impose competitive pressure on *B. purificata* in terms of food resources and habitat space (Zhang et al., 2017). Such environmental changes could induce a stress response in *B. purificata*, thereby altering its gut microbial structure (Wei et al., 2024). Alternatively, *B. purificata* may adjust its feeding habits to alleviate interspecific competition for limited food resources (Li J. et al., 2025; Tao et al., 2023), resulting in structural and functional adaptations of the gut microbiota to dietary shifts, which help maintain nutrient absorption and intestinal homeostasis (Zhao et al., 2025). It is thus inferred that the significant shift in the microbial community structure of *B. purificata* may represent a responsive adaptation to the influence of *P. canaliculata* invasion (Givens et al., 2015).

Furthermore, the significant differences in gut microbial community structure observed between 30-day and 60-day monoculture groups may reflect functional changes in the gut microbiota associated with snail growth and developmental stages (Liu et al., 2022). This finding is consistent with numerous studies reporting that the intestinal microbiota of aquatic animals undergoes dynamic successional changes throughout development (Yan et al., 2012).

### 4.5 Impact of *Pomacea canaliculata* stress on gut microbial composition in *Bellamya purificata*

At the phylum level, no significant differences were observed in the relative abundance of gut microbiota between monoculture and co-culture groups of *B. purificata* at 30 days. In contrast, at 60 days, the co-culture group showed significantly higher relative abundances of *Bacteroidota*, *Actinobacteriota*, and *Planctomycetota* compared to the monoculture group (*p* < 0.05). *Bacteroidota* and *Actinobacteriota* are considered beneficial bacterial phyla in the intestinal tracts of aquatic animals. *Bacteroidota* contributes to host growth by efficiently utilizing low-carbon compounds (Xu et al., 2024), while *Actinobacteriota* produces antibacterial substances and nutritional factors that suppress pathogenic bacteria and promote growth in aquaculture species (James et al., 2023; Liu M. Q. et al., 2025). *Planctomycetota*, commonly found in the intestinal tracts of aquatic animals, water environments, and sediments, plays an important role in nitrogen cycling (Huang et al., 2024).

At the genus level, after 30 days of cultivation, the co-culture group of *B. purificata* showed a considerably higher relative abundance of *Acinetobacter* but dramatically lower relative abundances of *Pseudomonas*, *Shewanella*, *Edwardsiella*, *Candidatus_Bacilloplasma*, and *Candidatus_Hepatoplasma* compared to the monoculture group. Most species of the genus *Acinetobacter* have been characterized as pathogens. Their increased presence in the gut has been linked to morphological changes in endocrine cells and a state of nutrient insensitivity, potentially even contributing to metabolic diseases (Wang et al., 2023). *Pseudomonas*, *Shewanella*, *Edwardsiella*, and *Acinetobacter* are commonly present in the intestines of healthy aquatic animals (Wang, 2021; Liu et al., 2024; Chen, 2024). Many species within *Pseudomonas* and *Shewanella* have been identified as potential probiotics and are used in aquaculture (Wu et al., 2012; Jiang, 2020). For example, supplementing *grass carp* feed with *Pseudomonas monteilii* JK-1 has been shown to increase body weight, improve specific growth rate and survival, reduce pathogen load, and enhance the expression of immune-related genes and the activity of antioxidant enzymes (Qi et al., 2024). Similarly, adding *Rhodopseudomonas palustris* to the diet of *Penaeus japonicus* greatly enhances immune and antioxidant levels, alleviating stress induced by pesticide residues (Li, 2024). Supplementing Pacific white shrimp feed with *Shewanella* notably improves weight gain and specific growth rate, induces the expression of antioxidant- and immune-related genes, enriches beneficial gut bacteria, and reduces the abundance of potential pathogens (Wei et al., 2021). The functional roles of *Candidatus_Bacilloplasma* and *Candidatus_Hepatoplasma* remain poorly understood. Some studies suggest that the relative abundance of *Candidatus_Bacilloplasma* is significantly higher in fast-growing shrimp populations compared to slow-growing ones (Imaizumi et al., 2022); while sarvation has been observed to reduce its abundance in river crabs, which recovers upon refeeding (Wang et al., 2019), implying a potential role in growth and metabolism. Few studies have focused on the influence of *Candidatus_Hepatoplasma* in aquatic animals, though its abundance in crab hepatopancreas is highly correlated with hepatopancreatic necrosis disease (Shen et al., 2017). In terrestrial isopods, *Candidatus_Hepatoplasma* is generally thought to enhance host survival under low-nutrient conditions (Leclercq et al., 2014).

After 60 days of cultivation, the co-culture group of *B. purificata* exhibited decidedly higher relative abundances of *Aeromonas*, *Cloacibacterium*, *Kurthia*, *Planktothrix*_NIVA-CYA_15, *Gluconobacter*, *Microseira_Carmichael-Alabama*, and *Methyloparacoccus*, while the relative abundances of *Enterobacter*, *Enterococcus*, *Candidatus_Hepatoplasma*, *LactoBacillus*, and *Akkermansia* were markedly lower compared to the monoculture group. *Aeromonas* and *Enterobacter* showed the most notable differences between monoculture and co-culture groups. Both are core microbiota in the gut tract of *B. purificata*, and many species within these genera have been identified as pathogen (Kang et al., 2018; Shi et al., 2021; Cui et al., 2022). In contrast, the other mentioned bacterial groups have not been reported to cause diseases in aquatic animals and are largely considered beneficial. *Cloacibacterium*, a core microbe in freshwater gastropods, can be vertically transmitted among hosts, promoting adaptive evolution and supporting host growth and health (Lin et al., 2023). *Gluconobacter* participates in the oxidation of various sugars and polyols, facilitating energy cycling within the gut microbial community. *Enterococcus* contributes to enhanced carbohydrate and protein metabolism, non-specific immune enzyme activity, and disease resistance in the host (Satish et al., 2011; Ju et al., 2018). *LactoBacillus* inhibits the growth of pathogenic microorganisms and strengthens digestive enzyme and non-specific immune enzyme activities (Zhang et al., 2017; Shui et al., 2021; Zhang et al., 2024). *Akkermansia* promotes intestinal mucus secretion, modulates mucosal barrier function, helps regulate blood glucose levels, and maintains glucose homeostasis (Liu Y. J. et al., 2025). Additionally, *Planktothrix*_NIVA-CYA_15 and *Microseira_Carmichael-Alabama* are common *Cyanobacteria*. The former is often abundant in eutrophic water bodies, and the latter proliferates in phosphorus-enriched sediments (Pancrace et al., 2017; Anderson et al., 2019). While the OTU clustering method employed in this study is suitable for detecting community changes at higher taxonomic levels, its resolution for distinguishing between strains within highly diverse genera is limited. Therefore, the observed changes in relative abundance at the genus level should be interpreted as indicators of broader microbial community restructuring under invasion pressure, rather than as definitive evidence for the increase or decrease of specific functional groups.

At 30 days of cultivation, stress from *P. canaliculata* led to a significant increase in *Acinetobacter* within the gut microbiota of *B. purificata*, which may contribute to metabolic disorders in this species. Concurrently, the relative abundances of several probiotic bacteria decreased, indicating a notable negative impact of co-culture with *P. canaliculata* on *B. purificata* at this stage. By 60 days of cultivation, the relative abundances of both pathogenic and beneficial bacteria in the gut microbiota of *B. purificata* showed simultaneous increases and decreases. This pattern of change may be attributed to multiple factors including the environment, diet, and host growth. The results indicate that the stress from *P. canaliculata* still exerted some influence on *B. purificata*, though its adverse effects were less pronounced. Some of the observed shifts further suggest that *B. purificata* may have developed a degree of adaptation to the stress imposed by *P. canaliculata*. For instance, the increased relative abundance of *Cloacibacterium* may have promoted adaptive evolution in *B. purificata* (Lin et al., 2023). Shifts in dietary composition, such as increased proportions of *Chroococcus*, *Stauroneis*, and Pediastrum, and decreased proportions of *Cymbella* and *Oscillatoria*—suggest altered feeding strategies in response to food competition with *P. canaliculata*. Furthermore, the increased abundance of *Methyloparacoccus*, which uses methane as its sole carbon source (Deng et al., 2015), may reflect metabolic adaptations to energy resource competition.

At both the 30-day and 60-day culture periods, the relative abundance of *Euglena* in the intestine of *B. purificata* in the co-culture group was higher than that in the monoculture group. Although *Euglena* is considered a beneficial alga that can activate the immune system, promote beneficial bacteria, and inhibit pathogens (Rodríguez-Zavala et al., 2010; Suzuki et al., 2018), its relative abundance in this study showed a significant positive correlation with the relative abundance of the conditional pathogen *Aeromonas*. Existing studies have confirmed that the efficacy of prebiotics is closely related to the physiological state of the host (Hashemitabar and Hosseinian, 2024; Zhang et al., 2024). For instance, probiotics that enhance the immunity of rainbow trout under 20 °C heat stress become ineffective under the more severe stress of 25 °C (Nakhei et al., 2023). It is therefore hypothesized that stress induced by *P. canaliculata* on *B. purificata* alters the intestinal microenvironment, leading to an anomalous relationship between the abundances of *Euglena* and *Aeromonas*.

### 4.6 Impact of *Pomacea canaliculata* stress on gut microbial function in *Bellamya purificata*

PICRUSt2 analysis revealed that metabolism-related pathways accounted for the highest proportion of enriched functional genes in the intestinal microbiota of both *P. canaliculata* and *B. purificata*, which is consistent with the findings reported by Li Han in different geographic populations of *Quasipaa spinosa* (Li et al., 2025b). This suggests that the gut microbiota may play an important role in the digestion and metabolism of complex biochemical substances within the intestine.

At 30 days of cultivation, the co-culture group of *B. purificata* exhibited appreciably higher enrichment in pathways associated with neurodegenerative diseases, and significantly lower enrichment in immune diseases and signaling molecules and interaction pathways compared to the monoculture group. This implies that short-term stress from *P. canaliculata* might negatively affect might negatively affect the neural and immune functions in *B. purificata*. In contrast, the co-culture group of *B. purificata* at 60 days showed considerably higher enrichment in pathways related to cell motility, cellular community – prokaryotes, and the digestive system compared to the 30-day group. These observations suggest that prolonged stress from *P. canaliculata* could be associated with enhances the motility and digestive intensity of the intestinal microbiota in *B. purificata*, potentially representing an adaptive strategy to improve competitive advantage during development.

Overall, these predictive functional results indicate short-term stress from *P. canaliculata* may have adverse effects on *B. purificata*, while long-term stress appears to be linked to regulatory and adaptive responses in its intestinal microbiota. It should be noted that these interpretations are derived from *in silico* predictions, and further experimental validation is needed to confirm the associated phenotypic and physiological implications.

## 5 Conclusion

At 30 days of cultivation, the co-culture group of *B. purificata* showed no significant differences in gut microbial abundance or community structure compared to the monoculture group, but exhibited greatly higher diversity and an increased relative abundance of *Acinetobacter*. Meanwhile, the relative abundances of several beneficial bacterial genera were significantly reduced. Functional gene analysis revealed considerably higher enrichment in pathways associated with neurodegenerative diseases, and dramatically lower enrichment in those related to immune diseases and signaling molecule interactions.

By 60 days, although microbial abundance and diversity did not differ appreciably between culture modes, microbial community structure had diverged markedly. The co-culture group showed decidedly higher relative abundances of *Bacteroidota*, *Actinobacteriota*, *Planctomycetota*, *Aeromonas*, and *Cloacibacterium*, while the abundances of *Enterobacter* and *Enterococcus* were markedly lower. Gene enrichment analysis showed significantly increased activity in pathways involved in cell motility and cellular community-prokaryotes.

In summary, under the stress of *P. canaliculata*, the structure and predicted functions of the gut microbiota in *B. purificata* exhibited distinct response patterns at 30 and 60 days. These temporal changes suggest that its gut microbial may have some adaptive regulation in response to sustained biological stress.

## Data Availability

The data presented in this study are publicly available. The data can be found at: https://www.ncbi.nlm.nih.gov, PRJNA1358984.

## Footnotes

1
http://drive5.com/uchime/uchime_download.html
